# High proportion of knowlesi malaria in recent malaria cases in Malaysia

**DOI:** 10.1186/1475-2875-13-168

**Published:** 2014-05-03

**Authors:** Ruhani Yusof, Yee Ling Lau, Rohela Mahmud, Mun Yik Fong, Jenarun Jelip, Hie Ung Ngian, Sahlawati Mustakim, Hani Mat Hussin, Noradilah Marzuki, Marlindawati Mohd Ali

**Affiliations:** 1 Department of Parasitology, Faculty of Medicine, Tropical Infectious Disease Research and Education Center (TIDREC), University of Malaya, 50603 Kuala Lumpur, Malaysia; 2 Sabah State Health Department, Tingkat 1, 3 & 6, Rumah Persekutuan, Jalan Mat Salleh, 88590 Kota Kinabalu, Sabah, Malaysia; 3 Hospital Kapit, Jalan Mamora, 96800 Kapit, Sarawak, Malaysia; 4 Microbiology Laboratory, Department of Pathology, Hospital Tengku Ampuan Rahimah, Jalan Langat, 41200 Klang, Selangor, Malaysia; 5 Kelantan State Health Department, Aras 5, Wisma Persekutuan, Jalan Bayam, 15590 Kota Bharu, Kelantan, Malaysia; 6 Department of Pathology, Hospital Kuala Lipis, 27200 Kuala Lipis, Pahang, Malaysia; 7 Department of Pathology, Hospital Tuanku Ja’afar Jalan Rasah, 70300 Seremban, Negeri Sembilan, Malaysia

**Keywords:** Malaria distribution, *Plasmodium knowlesi*, Malaysia Borneo, Peninsular Malaysia, Nested PCR, Microscopy, SSU rRNA gene

## Abstract

**Background:**

*Plasmodium knowlesi* is a simian parasite that has been recognized as the fifth species causing human malaria. Naturally-acquired *P. knowlesi* infection is widespread among human populations in Southeast Asia. The aim of this epidemiological study was to determine the incidence and distribution of malaria parasites, with a particular focus on human *P. knowlesi* infection in Malaysia.

**Methods:**

A total of 457 microscopically confirmed, malaria-positive blood samples were collected from 22 state and main district hospitals in Malaysia between September 2012 and December 2013. Nested PCR assay targeting the 18S rRNA gene was used to determine the infecting *Plasmodium* species.

**Results:**

A total of 453 samples were positive for *Plasmodium* species by using nested PCR assay. *Plasmodium knowlesi* was identified in 256 (56.5%) samples, followed by 133 (29.4%) cases of *Plasmodium vivax*, 49 (10.8%) cases of *Plasmodium falciparum*, two (0.4%) cases of *Plasmodium ovale* and one (0.2%) case of *Plasmodium malariae*. Twelve mixed infections were detected, including *P. knowlesi*/*P. vivax* (n = 10), *P. knowlesi*/*P. falciparum* (n = 1)*,* and *P. falciparum*/*P. vivax* (n = 1). Notably, *P. knowlesi* (Included mixed infections involving *P. knowlesi* (*P. knowlesi*/*P. vivax* and *P. knowlesi* /*P. falciparum*)) showed the highest proportion in Sabah (84/115 cases, prevalence of 73.0%), Sarawak (83/120, 69.2%), Kelantan (42/56, 75.0%), Pahang (24/25, 96.0%), Johor (7/9, 77.8%), and Terengganu (4/5, 80.0%,). In contrast, the rates of *P. knowlesi* infection in Selangor and Negeri Sembilan were found to be 16.2% (18/111 cases) and 50.0% (5/10 cases), respectively. Sample of *P. knowlesi* was not obtained from Kuala Lumpur, Melaka, Perak, Pulau Pinang, and Perlis during the study period, while a microscopically-positive sample from Kedah was negative by PCR.

**Conclusion:**

In addition to Sabah and Sarawak, which have been known for high prevalence of *P. knowlesi* infection, the findings from this study highlight the widespread distribution of *P. knowlesi* in many Peninsular Malaysia states.

## Background

Malaria is one of the most widespread diseases in the world and is endemic throughout tropical and subtropical regions. In 2012, there were an estimated 207 million cases of malaria, causing approximately 627,000 deaths [1]. Moreover, it is thought that as many as 3.4 billion people are at risk for malarial infection, mostly in Africa and Southeast Asia.

Five *Plasmodium* species are known to infect humans: *Plasmodium falciparum, Plasmodium vivax, Plasmodium malariae, Plasmodium ovale*, and *Plasmodium knowlesi*[2]. *Plasmodium falciparum* causes the most severe disease and tends to predominate in tropical areas, whereas *P. vivax* is the predominant species outside of Africa.

Malaria constitutes an important public health problem in Malaysia, especially in the states of Sabah and Sarawak, in Borneo island as well as the central interior regions of Peninsular Malaysia (i e, the shared borders of Perak, Pahang and Kelantan), which have a predominantly aboriginal populations. In 2012, there were 4725 cases of malaria including *P. falciparum* (n = 897, 19%)*, P. vivax* (n = 1,458, 30.9%), *P. malariae* (n = 485, 10.3%), *P. knowlesi* (n = 1813, 38.4%)*, P. ovale* (n = 8, 0.1%)*,* mix-infection (n = 64, 1.35%) and 12 (0.25%) malaria-related deaths in Malaysia [1].

*Plasmodium knowlesi* is a malaria parasite infecting Old World monkeys that is prevalent in Southeast Asia [3]. It has been recognized as the fifth medically important malaria parasite [4]. A large number of naturally acquired human *P. knowlesi* infections were initially reported in the Kapit Division of Sarawak (Malaysia) [5], which were followed by cases in several geographical regions of Southeast Asia. Previous epidemiological studies have revealed that *P. knowlesi* infections occur in Sarawak and Sabah [5,6], Kalimantan, Indonesian Borneo [7], Peninsular Malaysia [8,9] Thailand [10,11], Singapore [12], Philippines [13], Vietnam [14], and Myanmar [15]. These infections occur in forested areas, which is consistent with exposure to the *Anopheles* species suspected to be responsible for *P. knowlesi* transmission such as *Anopheles hackeri*, *Anopheles balabacensis*, *Anopheles cracens* and *Anopheles latens*[16-18].

*Plasmodium knowlesi* carries high potential of causing severe or fatal diseases [9]. However, this potentially life-threatening parasite has been frequently misdiagnosed as *P. malariae* via conventional microscopy due to similarities in morphology at certain asexual stages. Indeed, microscopic examination of the asexual stages of *Plasmodium* on thick and thin blood film is unable to distinguish early trophozoites of *P. knowlesi* from *P. falciparum* and late erythrocytic stages from *P. malariae*[5]. Nevertheless, microscopy represents the most commonly used method for detecting malaria parasites and has been considered as the gold standard for diagnosis, especially for detection and management of early cases in Malaysia. However, the precision and reliability of microscopy depends largely on the quality of stained blood films, parasite density and microscopist expertise. Furthermore, it is difficult to detect mixed infections or low parasitaemia using this conventional method, especially for cases involving *P. knowlesi*. Thus, in the present study, molecular approach was employed to investigate the epidemiology of *P. knowlesi* in Malaysia. PCR assay has been described as the most sensitive and specific technique for malarial detection and is capable of identifying mixed infections using species-specific primers [19,20].

## Methods

### Study sites and sample collection

**Figure 1:**
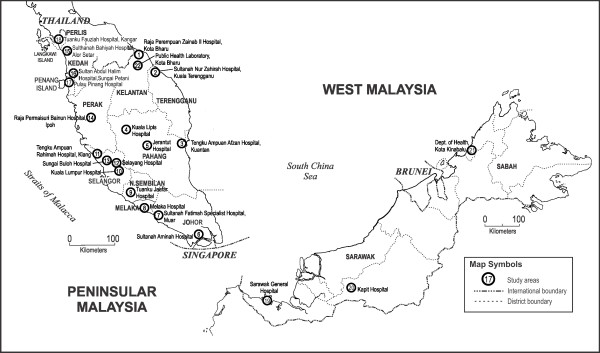
Map of Malaysia showing location of the study areas.

This study was approved by the Medical Research Ethic Committee (MREC) Ministry of Health Malaysia and Medical Ethics Committee of University Malaya Medical Centre. This study was conducted at 22 state and district government hospitals all over Malaysia: Raja Perempuan Zainab II Hospital, Kota Bharu; Sultanah Nur Zahirah Hospital, Kuala Terengganu, Terengganu; Tengku Ampuan Afzan Hospital, Kuantan, Pahang; Kuala Lipis Hospital, Kuala Lipis, Pahang; Jerantut Hospital Jerantut, Pahang; Sultanah Aminah Hospital, Johor Bahru, Johor; Sultanah Fatimah Specialist Hospital, Muar, Johor; Melaka Hospital, Melaka; Tuanku Jaafar Hospital, Seremban, Negeri Sembilan; Tengku Ampuan Rahimah Hospital, Klang, Selangor; Selayang Hospital, Selangor; Sungai Buloh Hospital, Selangor; Raja Permaisuri Bainun Hospital, Ipoh, Perak; Sultanah Bahiyah Hospital, Alor Setar, Kedah; Sultan Abdul Halim Hospital, Sungai Petani, Kedah; Pulau Pinang Hospital, Pulau Pinang; Tuanku Fauziah Hospital, Kangar, Perlis; Sarawak General Hospital, Kuching, Sarawak; Kapit Hospital, Kapit, Sarawak; Kota Bharu Public Health Laboratory, Kota Bharu, Kelantan and the Department of Vector Borne Disease Control Programme, Kota Kinabalu, Sabah (Figure 1).

Four-hundred and fifty-seven microscopically positive malaria blood samples (in EDTA tube), blood spots (drop on 903 Protein Saver Card; Whatman Ltd, NJ, USA) and blood films were collected from the above locations. Microscopic examination of Giemsa-stained thick and thin blood films were carried out by microscopists of the respective hospitals who have no knowledge of the PCR results. Positive malaria samples were sent by mail express to the Department of Parasitology, Faculty of Medicine, University of Malaya. No randomization was applied to the sampling. Due to logistical, as well as personal co-operative difficulties, the original intention of complete recruitment of all malaria patients was not achieved. Nevertheless, sampling encompassing nine states of Malaysia was successfully done.

### DNA extraction

Templates DNA for nested PCR assay were prepared. DNA extraction was performed on 100 μl blood collected in EDTA-vacutainer (n = 352) using DNeasy Blood & Tissue Kit (Qiagen, Germany). DNA extraction from dried blood spots (n = 81) and blood smears (n = 24) were conducted according to protocols described previously [21,22] to obtain 100 μl of purified template DNA. Purified DNA was stored at -20°C.

### Nested PCR assay

The species of the malaria parasite in the samples was determined by nested PCR assay. This assay targets the *Plasmodium* small subunit ribosomal RNA (SSU rRNA) gene. The primers used for the nested PCR assay were identical to those previously published [5]. The reaction mixture for the first PCR step had 2 μl of DNA template, 250 nmol/L of each primer (rPLU 1 and rPLU 5), PCR buffer (50 mmol/L KCl, 10 mmol/L Tris-HCl), 200 mmol/L of each deoxynucleoside triphosphate, 1.25 units of Taq DNA polymerase (Promega), and nuclease free water to a final volume of 25 μl. Primary amplification conditions were 94°C for 4 min; 35 cycles at 94°C for 30 sec, annealing at 55°C for 1 min, extension at 72°C for 1 min; and final extension at 72°C for 4 min. Two μl of the first amplification product was used as DNA template for each of the 20 μl secondary amplifications. The conditions and concentrations of the secondary amplification were identical to those of the primary except for the annealing temperature of 58°C for species-specific primers (rFAL1/rFAL2, rVIV1/rVIV2, rMAL1/rMAL2, Pmk8/Pmkr9 and rOVA1/rOVA4) and the amount of Taq Polymerase being 0.5 units. The product was analyzed by agarose gel electrophoresis. All PCR *P. knowlesi* positive samples were sent to Mytacg Bioscience for DNA sequencing to confirm the species.

### Statistical analysis

Statistical analysis was carried out using the SPSS software programme for Windows version 17 (SPSS, Chicago, IL, USA). A Pearson’s Chi-square test was used to test the association between each variable and the confidence interval (95% CI) was calculated to explore the association between variables of interest. The level of statistical significance was set at p <0.05 for each test.

## Results

A total of 453 out of 457 samples were positive for *Plasmodium* sp*.* based on nested PCR assay (Table 1). Among the *Plasmodium* species identified, *P. knowlesi*, *P. vivax, P. falciparum, P. ovale*, and *P. malariae* were responsible for 56.5% (n = 256), 29.4% (n = 133), 10.8% (n = 49), 0.4% (n = 2), 0.2% (n = 1), respectively. Moreover, 12 mixed infections were detected, including *P. knowlesi*/*P. vivax* (n = 10), *P. knowlesi*/*P. falciparum* (n = 1), and *P. falciparum*/*P. vivax* (n = 1).

**Table 1:** PCR-based species profile of malaria cases collected from areas under study

**States**	**Number of malaria-positive samples by PCR (%, 95% CI)**								**Total**
	**Pf**	**Pk**	**Pv**	**Pm**	**Po**	**Pv + Pk**	**Pf + Pk**	**Pf + Pv**	
Sarawak	11	80	25	0	1	3	0	0	120
	(9.2, 6.6–11.8)	(66.7, 62.4–71.0)	(20.8, 17.1–24.5)	(0)	(0.8,–0.02–1.62)	(2.5, 1.1–3.9)	(0)	(0)	(100)
Sabah	14	82	15	1	0	1	1	1	115
	(12.2, 9.2–15.2)	(71.3, 67.1–75.4)	(13.0, 9.9–16.1)	(0.9, 0.04–1.7)	(0)	(0.9, 0.04–1.7)	(0.9, 0.04–1.7)	(0.9, 0.04–1.7)	(100)
Selangor	18	16	74	0	1	2	0	0	111
	(16.2, 12.8–19.6)	(14.4, 11.2–17.6 )	(66.7, 62.4–71.0)	(0)	(0.9, 0.04–1.7)	(1.8, 0.6–3.0)	(0)	(0)	(100)
Kelantan	4	40	10	0	0	2	0	0	56
	(7.1, 4.7–9.5)	(71.4, 67.2–75.5)	(17.9, 14.4–21.4)	(0)	(0)	(3.6, 1.9–5.3)	(0)	(0)	(100)
Pahang	0	24	1	0	0	0	0	0	25
	(0)	(96.0, 94.2–97.8 )	(4.0, 2.2–5.8)	(0)	(0)	(0)	(0)	(0)	(100)
Negeri Sembilan	1	4	4	0	0	1	0	0	10
	(10.0, 7.2–12.8)	(40.0, 35.5–44.5)	(40.0, 35.5–44.5)	(0)	(0)	(10.0, 7.2–12.8)	(0)	(0)	(100)
Johor	0	6	2	0	0	1	0	0	9
	(0)	(66.7, 62.4–71.0)	(22.2,18.4–26.0)	(0)	(0)	(11.1, 8.2–14.0)	(0)	(0)	(100)
Kedah	1	0	1	0	0	0	0	0	2
	(50.0, 45.4–54.6)	(0)	(50.0, 45.4–54.6)	(0)	(0)	(0)	(0)	(0)	(100)
Terengganu	0	4	1	0	0	0	0	0	5
	(0)	(80.0, 68–72)	(20.0, 16.3–23.7)	(0)	(0)	(0)	(0)	(0)	(100)
**Total**	49	256	133	1	2	10	1	1	453
	(10.8, 7.2–13.7 )	(56.5, 51.9–61.1)	(29.4, 25.2–33.6)	(0.2, –0.2–0.6)	(0.4,-.0.18- 0.98)	(2.2, 0.9–3.6)	(0.2 ,–0.2–0.6)	(0.2 , –0.2–0.6)	(100)

Pf = *P. falciparum*; Pk = *P. knowlesi*; Pv = *P. vivax*; Pm = *P. malariae*; Po = *P.ovale*; Pv + Pk = *P. vivax* and *P. knowlesi*; Pf + Pk = *P. falciparum* and *P. knowlesi*; Pf + Pv = *P. falciparum* and *P. vivax*.

**Figure 2:**
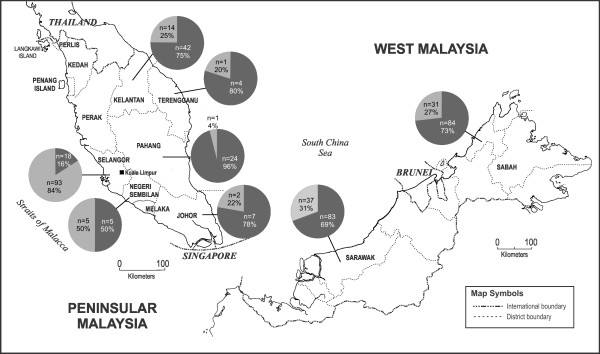
**Map of Malaysia showing location of the positive *Plasmodium knowlesi *areas.** Pie charts represent number of cases and percentages of *P. knowlesi* (black) and non-*P. knowlesi* (grey) malaria infections determined by species-specific nested PCR.

Notably, *P. knowlesi* (included mixed infections involving *P. knowlesi* (*P. knowlesi*/*P. vivax* and *P. knowlesi*/*P. falciparum*) showed the high prevalence in Sabah (84 out of 115 cases, proportion of 73.0%), Sarawak (83 out of 120 cases, 69.2%), Kelantan (42 out of 56 cases, 75.0%), Pahang (24 out of 25 cases, 96.0%), Johor (7 out of 9 cases, 77.8%), Terengganu (4 out of 5 cases, 80.0%). In contrast, the rates of *P. knowlesi* infection in Selangor and Negeri Sembilan were 16.2% (18 out of 111 cases) and 50.0% (5 out of 10 cases), respectively. Sample of *P. knowlesi* was not obtained from Kuala Lumpur, Melaka, Perak, Kedah, Pulau Pinang, and Perlis during the study period, while a microscopically-positive sample from Kedah was negative by PCR (Figure 2).

Among the *Plasmodium*-positive samples, 56.5% (256 out of 453) were infected with *P. knowlesi* only, whereas 11 displayed mixed infections involving *P. knowlesi* (10 *P. knowlesi*/*P. vivax* and 1 *P. knowlesi*/*P. falciparum*). Many cases of single *P. knowlesi* infection had been microscopically misdiagnosed as other species, including *P. malariae* (n = 72, 28.1%), *P. vivax* (n = 8, 3.1%), and *P. falciparum* (n = 3, 1.2%). Furthermore, 6 mixed infections involving *P. knowlesi* (with either *P. falciparum* or *P. vivax*) were misdiagnosed microscopically as single *P. falciparum* (n = 3), single *P. vivax* (n = 2), or single *P. malariae* (n = 1) infections and five cases of mixed infection (i.e. *P. knowlesi*/*P. vivax*) were misdiagnosed microscopically as single *P. knowlesi* (Table 2).

**Table 2:** **Detection of *Plasmodium *** **species by microscopy and polymerase chain reaction (PCR) methods in 453 patients with malaria in Malaysia**

	**Result of microscopic examination**							
**PCR results**	***Pf***	***Pk***	***Pv***	***Pm***	***Po***	***Pk + Pv***	***Pf + Pm***	**No. of cases by PCR**
*P. falciparum*	46	1	1	1	0	0	0	49
*P. vivax*	0	3	122	6	1	1	0	133
*P. malariae*	0	0	1	0	0	0	0	1
*P. ovale*	0	0	2	0	0	0	0	2
*P. knowlesi*	3	172	8	72	0	0	1	256
*P. knowlesi* and *P. vivax*	2	5	2	1	0	0	0	10
*P. knowlesi* and *P. falciparum*	1	0	0	0	0	0	0	1
*P. falciparum* and *P. vivax*	0	0	1	0	0	0	0	1
**No. of cases by microscopy**	52	181	136	81	1	1	1	453

Note: Pf = *P. falciparum*; *Pk* = *P. knowlesi*; *Pv* = *P. vivax*; *Pm* = *P. malariae*; *Po* = *P.ovale*; *Pv + Pk* = *P. vivax* and *P. knowlesi*; *Pf + Pm* = *P. falciparum* and *P. falciparum.*

In addition, the PCR method revealed that six cases of *P. vivax* and one case of dangerous *P. falciparum* had been wrongly identified as *P. malariae*, which is less threatening. Also, two cases of *P. ovale* infection were considered to be *P. vivax* by microscopy, whereas a case of *P. vivax* was considered to be *P. ovale*. Other than that, cases of *P. falciparum* and *P. vivax* (one each) were diagnosed as *P. knowlesi*. Notably there were other cases of single *P. knowlesi* and *P. vivax* had been wrongly identified as mixed infection; *P. falciparum/P. malariae* and *P. knowlesi*/*P. vivax* respectively (Table 2). However in this study, four microscopically malaria-positive cases (one *P. falciparum* and three *P. knowlesi*) were negative when tested by nested PCR assay.

The age and gender distribution of malaria cases were characterized (Table 3). There was no significant association between age, gender and malaria reported cases. *Plasmodium knowlesi* infections were detected within all age groups of the study populations, with the youngest being six months old and the oldest being 82 years old (mean = 33.8 years; median = 30.0 years). The infection was more prevalent among adults (Table 4); the highest proportion (28.5%) was among patients between 21 and 30 years old. There was a significant difference (p = 0.008) detected among the age groups infected by *P. knowlesi* (Table 5). The percentage of male patients (77.9%) was higher compared to female patients (22.1%) (Table 6).

**Table 3:** Age- and gender-based distribution of malaria cases in the area under study

**States**	**Number of patients according to age groups (%)**										**Gender (%)**	
	**0–10**	**11–20**	**21–30**	**31–40**	**41–50**	**51–60**	**61–70**	**71–80**	**81–90**	**Total**	**Male**	**Female**
Sarawak	2	14	18	15	33	26	10	1	1	120	90	30
	(1.7)	(11.7)	(15.0)	(12.5)	(27.5)	(21.7)	(8.3)	(0.8)	(0.8)	(100.0)	(75.0)	(25.0)
Sabah	10	22	31	15	18	12	6	1	0	115	90	25
	(8.7)	(19.1)	(27.0)	(13.0)	(15.7)	(10.4)	(5.2)	(0.9)	(0)	(100.0)	(78.3)	(21.7)
Selangor	0	13	69	19	8	2	0	0	0	111	92	19
	(0)	(11.7)	(62.2)	(17.1)	(7.2)	(1.8)	(0)	(0)	(0)	(100.0)	(82.9)	(17.1)
Kelantan	2	5	27	11	10	1	0	0	0	56	43	13
	(3.6)	(8.9)	(48.2)	(19.6)	(17.9)	(1.8)	(0)	(0)	(0)	(100.0)	(76.8)	(23.2)
Pahang	0	1	8	9	6	1	0	0	0	25	19	6
	(0)	(4.0)	(32.0)	(36.0)	(24.0)	(4.0)	(0)	(0)	(0)	(100.0)	(76.0)	(24.0)
Negeri Sembilan	1	1	3	3	1	1	0	0	0	10	9	1
	(10.0)	(10.0)	(30.0)	(30.0)	(10.0)	(10.0)	(0)	(0)	(0)	(100.0)	(90.0)	(10.0)
Johor	0	1	1	6	0	0	1	0	0	9	8	1
	(0)	(11.1)	(11.1)	(66.7)	(0)	(0)	(11.1)	(0)	(0)	(100.0)	(88.9)	(11.1)
Kedah	0	1	1	0	0	0	0	0	0	2	2	0
	(0)	(50.0)	(50.0)	(0)	(0)	(0)	(0)	(0)	(0)	(100.0)	(100.0)	(0)
Terengganu	0	1	2	1	0	1	0	0	0	5	4	1
	(0)	(20.0)	(40.0)	(20.0)	(0)	(20.0)	(0)	(0)	(0)	(100.0)	(80.0)	(20.0)
**Total**	15	59	160	79	76	44	17	2	1	453	357	96
	(3.3)	(13.0)	(35.3)	(17.4)	(16.8)	(9.7)	(3.8)	(0.4)	(0.2)	(100.0)	(78.8)	(21.2)
*X*^*2*^, P	52,8, 0.597										10.6, 0.158	

**Table 4:** **Distribution of *Plasmodium knowlesi *** **according to age group (children and adult) diagnosed by PCR**

**Age (year)**	**Number**	**Percentage**
Children (0–14)	18	6.7%
Adult (>15 years)	249	93.3%
*X*^*2*^		0.15
P		0.903
*Total	267	100

*Including mixed infections involving *P. knowlesi.*

**Table 5:** **Distribution of *Plasmodium knowlesi *** **according to age group diagnosed by PCR**

**Age group**	**Number**	**Percentage**
0–10	10	3.7%
11–20	34	12.7%
21–30	76	28.5%
31–40	46	17.2%
41–50	53	19.9%
51–60	32	12%
61–70	14	5.2%
71–80	1	0.4%
81–90	1	0.4%
*X*^*2*^		20.8
P		0.008
Total	267	100

**Table 6:** **Distribution of *Plasmodium knowlesi *** **according to gender diagnosed by PCR**

**Gender**	**Number**	**Percentage**
Male	208	77.9%
Female	59	22.1%
*X*^*2*^		0.57
P		0.32
Total	267	100

## Discussion

Laboratory diagnosis of malaria in disease-endemic countries routinely relies on microscopic detection of parasites in blood smears. The discovery of *P. knowlesi* in humans, which is commonly misdiagnosed as *P. malariae* by microscopy due to morphological similarities, has contributed to a fundamental need for improved diagnostic methods for differentiating malaria parasites [9]. In this regard, *P. knowlesi*-specific primers [5] can be used to accurately identify *P. knowlesi* infections using nested PCR assays, which are more sensitive and specific than microscopy [20,23]. However, nested PCR is not yet appropriate for routine clinical use because it is expensive, time-consuming, and non-quantitative. In addition, it requires specialized equipment. Nevertheless, in the present study, the nested PCR technique was successfully used to determine recent incidence of *P. knowlesi* infections in Malaysia.

Recently, significant reduction in malaria cases has been observed and achieved in Malaysia [24]. Malaysia is now striving to be malaria-free in the Peninsular by 2015 and in Malaysian Borneo by 2020 [25]. However, recently, a significant increase in *P. knowlesi* cases was observed in Sabah between 2004 and 2011 and this trend threatens malaria elimination [26].

Based on preliminary study, it is indicated that *P. knowlesi* was accounted for more than half of all cases of malaria in Malaysia. Among the 256 single *P. knowlesi* infections, 72 (28.1%) had been microscopically misdiagnosed as *P. malariae*. PCR findings also demonstrated that *P. knowlesi* were detected in almost all cases of microscopically identified *P. malariae* infections. *P. knowlesi* cases that were microscopically misdiagnosed as *P. malariae* were not found in Pahang and Kelantan. Meanwhile, only three cases of *P. knowlesi* infection were misdiagnosed as *P. malariae* in Sarawak. However, most of the *P. knowlesi* infections in Sabah were misdiagnosed as *P. malariae* by microscopic examination. The decrease in microscopy diagnostic discrepancy is due to the increased awareness about *P. knowlesi* infections among the local laboratory technicians and microscopists. Coupled with the fact that *P. malariae* infection is not frequently found in Malaysia, the encounter of malaria parasites with morphology similar to that of *P. malariae* has been reported as “*P. knowlesi*” infection by default. In addition, clinical data such as the relatively high parasitaemia and low platelet reading serve as useful guides in diagnosing *P. knowlesi* infection as well.

From this study, it is clearly demonstrated that PCR represents a more sensitive and specific method for detecting mixed infections, which tend to be overlooked in the microscopy-based assay, especially when one of the species is present at a lower density. Moreover, PCR allows specific identification of *P. knowlesi*, which sometimes cannot be accurately diagnosed by microscopic examination. The negative nested PCR results yielded from the four microscopically malaria-positive cases were likely to be caused by DNA degradation of the samples. Coincidently, these four samples were DNA templates extracted from the blood smears, indicating the relatively inferior quality of DNA extracted from blood smears, as pointed out by previous studies [27,28]. Nevertheless, blood smears still serve as a good source of DNA extraction [22], especially for samples from isolated locations that are hard to reach.

The PCR primers (Pmk8 and Pmk9) used for *P. knowlesi* detection in this study were not the most recently developed primers. Issues regarding non-specific binding of these primers to *P. vivax* were reported previously [29]. Nevertheless, the specificity of PCR primer binding is influenced by many confounding factors and may vary from one laboratory to another, as well as from one PCR machine to another. For further confirmation of the nested PCR results, DNA sequencing on the PCR amplicons from primers rPLU 5 and rPLU 6 (size ~1200 bp) were conducted. Based on sequencing, all malaria-positive samples identified as *P. knowlesi*-positive by the Pmk8 and Pmkr9 primers were indeed *P. knowlesi*-infected samples.

From this study, patients ranged from young children to the elderly; however, this infection was more prevalent among adults, especially males. Only a small proportion of cases occurred in children; this may be related to the limited outdoor or forest activities by this group of patients. A previous study postulated that the transmission of *P. knowlesi* may occur from human to human due to the discovery of family clustering cases [30]. Nevertheless, no clustering of *P. knowlesi* cases was found in the present study. The infection was more prevalent among adults (Table 4) with a significant difference of p = 0.008 detected among the age groups (Table 5) infected with *P. knowlesi*. The highest rate of infection (28.5%) occured in patients in the age group of 21 to 30 (Table 5) who presumably are more active outdoors and more at risk of being bitten by an infected mosquito. This finding is in agreement with the inference stated by [31] that *P. knowlesi* infection is obtained from mosquito fed on infected monkey and not from humans.

Early *P. knowlesi* trophozoites (delicate ring) display morphological similarities to *P. falciparum*, while other blood stages are identical to *P. malariae*[5,9], resulting in misdiagnoses. In fact, the present study confirms that *P. knowlesi* could be misdiagnosed as *P. malariae* (majority), *P. falciparum, P. vivax*, or mixed infections (i e, *P. falciparum/P. malariae*). As *P. knowlesi* displays the shortest replication cycle of all known human and non-human primate malarias (24 hours), malaria caused by this species is potentially fatal [32]. Therefore, timely diagnosis and treatment are crucial for reducing the risk of complications. Unfortunately, misdiagnosis of potentially lethal *P. knowlesi* as *P. malariae* may result in delayed treatment [9]. For this reason, accurate identification of *Plasmodium* species is fundamental for proper clinical management and control strategies. In addition, any case demonstrating “*P. malariae*-like parasites” should be treated presumably as “*P. knowlesi* infection” while waiting for the molecular confirmation diagnosis.

## Conclusion

In addition to Sabah and Sarawak, which have high record of *P. knowlesi* infection, the present investigation highlights the widespread distribution of human knowlesi malaria in Peninsular Malaysia (Kelantan, Pahang, Selangor, Johor and Negeri Sembilan). In fact, *P. knowlesi* was found to be the most prevalent *Plasmodium* parasite infecting the population of Sabah and Sarawak (Malaysia Borneo) as well as Peninsular Malaysia. Accurate epidemiological data serve as an important foundation for developing effective control strategies for malaria. The present study has provided critical data that contribute to the better understanding on the distribution of *P. knowlesi* within the interior division of Sabah and Sarawak and central regions of Peninsular Malaysia.

## Consent

Consent was granted by patient/patient’s family for the publication of these case reports.

## Competing interests

The authors declare that they have no competing interests.

## Authors’ contributions

RY, JJ, HUG, SM, HMH, NM and MMA collected blood samples and patients’ data. RY, and YLL analyzed and interpreted the data. RY, YLL, RM and MYF arranged the data, conceptualized and prepared the manuscript. All authors read and approved the final manuscript.
